# Associations between the muscle quality index and adult lung functions from NHANES 2011–2012

**DOI:** 10.3389/fpubh.2023.1146456

**Published:** 2023-05-10

**Authors:** Luoqi Weng, Zhixiao Xu, Yuhan Chen, Chengshui Chen

**Affiliations:** ^1^Department of Pulmonary and Critical Care Medicine, The First Affiliated Hospital of Wenzhou Medical University, Wenzhou, China; ^2^Key Laboratory of Interventional Pulmonology of Zhejiang Province, The First Affiliated Hospital of Wenzhou Medical University, Wenzhou, China

**Keywords:** muscle quality index, lung function, NHANES, public health, cross-sectional research

## Abstract

**Background:**

The muscle quality index (MQI), as an important component of sarcopenia, is defined as the ratio of muscle strength to muscle mass. Lung function, is a clinical indicator to assess ventilation and air exchange function. This study investigated the relationship between lung function indices and MQI in the NHANES database from 2011 to 2012.

**Methods:**

This study included 1,558 adults from the National Health and Nutrition Examination Survey from 2011 to 2012. Muscle mass and muscle strength were assessed using DXA and handgrip strength, and all participants underwent pulmonary function measurements. Multiple linear regression and multivariable logistic regression were used to assess the correlation between the MQI and lung function indices.

**Results:**

In the adjusted model, MQI was significantly correlated with FVC% and PEF%. And, after quartiles of MQI in Q3, where FEV_1_%, FVC%, and PEF% were all associated with MQI, in Q4, a lower relative risk of a restrictive spirometry pattern was linked to increased MQI. Compared to the lower age group, the relationship between the MQI and lung function indices was more significant in the higher age group.

**Conclusion:**

There was an association between the MQI and lung function indices. Furthermore, in the middle-aged and older adult populations, lung function indicators and restrictive ventilation impairment were significantly associated with MQI. This implies that improving lung function through muscle training may be beneficial to this group.

## 1. Introduction

To our knowledge, sarcopenia is a condition that causes gradual loss of muscle strength and muscle mass. It always affects older adults and those suffering from chronic disease (1). Additionally, it can lead to falls, weakness, disability, functional impairment and even death (2). Recently, some research has shown that expiratory and inspiratory muscle strength is known to decline with age and may result from the decrease of elastic retraction and thorax compliance, which leads to the decline of some lung functions, such as forced expiratory volume in the first 1.0 s (FEV_1_), forced vital capacity (FVC) and peak expiratory flow rate (PEF) (3, 4). Furthermore, another study has presented that the decrease in muscle mass and strength is related to the rapid decline in lung functions (5). The components of lung function tests are very important tools in the clinical evaluation of respiratory health and disease. The main indicators of pulmonary ventilation include FEV_1_/FVC, FEV_1_, FVC, and PEF. An obstruction in airflow is defined as a ratio of less than 0.70 of forced expiratory volume in the 1.0 s (FEV_1_) to FVC, with FVC < 80% of predicted representing restrictive ventilatory dysfunction. FEV_1_ determines the severity of ventilatory function, whether it is obstructive, restrictive or mixed (6). PEF reflects respiratory muscle strength and changes in large airway caliber (7). Also, FENO, nitric oxide (NO) in the exhaled breath of humans, is important as a potentially useful non-invasive indicator of airway inflammation for the diagnosis and treatment of different airway inflammatory diseases (8, 9). Sarcopenia is an important syndrome of respiratory disease and is closely associated with poor outcomes. Early assessment of systemic body muscle strength and mass can be used to identify early lung function decline and high-risk healthy individuals developing chronic respiratory disease. For example, chronic obstructive pulmonary disease (COPD), which is a disease involving airflow limitation and persistent respiratory. In addition, COPD, a systemic disease, that can lead to muscle atrophy and dysfunction (10, 11).

The muscle quality index (MQI), defined as the ratio of muscle strength to muscle mass, is calculated by the handgrip strength (HGS) and the appendicular skeletal muscle mass (ASM) (12). ASM is usually assessed by dual-energy X-ray absorptiometry (DXA), a non-invasive instrument widely used in clinical practice to determine muscle mass (13). HGS is used to evaluate muscle strength and can be easily assessed by handgrip dynamometer, Moreover, HGS has been shown to correlate with whole-body muscle strength and can be combined with body composition to determine MQI (14, 15). Since ASM and HGS are easily available in clinical situations, we chose to use muscle quality (MQI) to access muscle condition. From what is known, no one has studied the relationship between the MQI and lung functions, and this is our initial attempt.

In the current study, we examined into how the MQI and lung function indices from the NHANES 2011–2012 correlated. We focused on exploring the correlation between MQI and different lung function indices (FEV_1_/FVC, FEV_1_%, FCV%, PEF%, FENO and obstructive or restrictive spirometry pattern) and performed restricted cubic splines, as well as exploring the differences in the associations between MQI and lung function indices in different age groups.

## 2. Materials and methods

### 2.1. Study population

Using a sophisticated, stratified, multistage sampling strategy, the National Health and Nutrition Examination Survey (NHANES) is a population-based, national cross-sectional survey. The primary target population is the noninstitutionalized civilian residing in the US who underwent laboratory assessment, physical examination and questionnaire survey related to nutrition and health. The conduct of NHANES was approved by the Ethics Review Board of the National Centre for Health Statistics and written informed consent was given by each participant.

**Figure 1:**
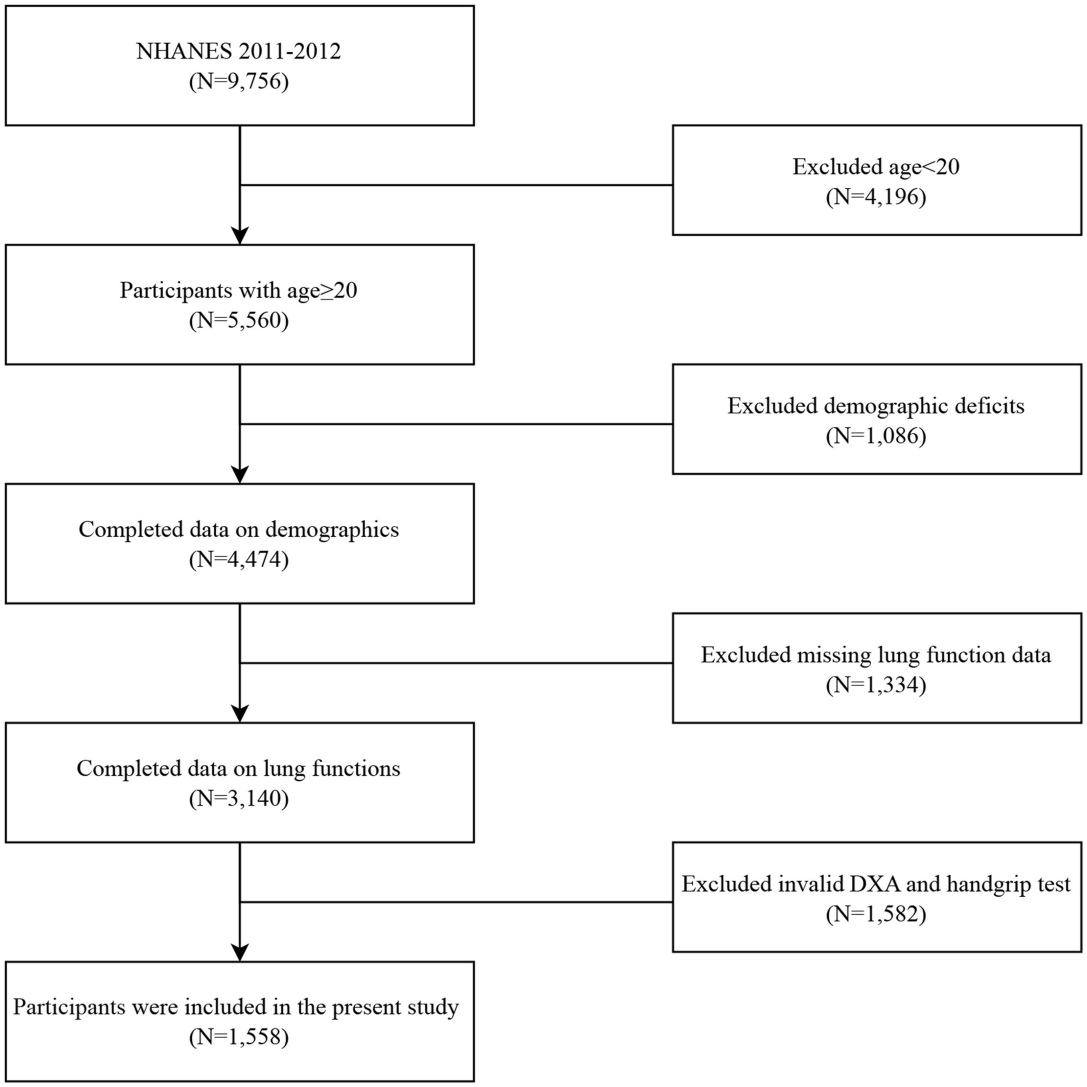
Flow chart of this study.

In this study, we used data from the NHANES collected from 2011 to 2012. The flow chart for inclusion and exclusion is shown in Figure 1. Among the 9,756 participants in the NHANES 2011–2012, individuals were excluded if (1) they were aged less than 20 years (*n* = 4,196), (2) they lost data for basic demographic data, including gender, age, race, etc. (*n* = 1,086), (3) they lost data for any spirometry data, or the quality of spirometry was unacceptable (*n* = 1,334), (4) they lost data for DXA and handgrip tests (*n* = 1,582). Finally, a total of 1,588 individuals were recruited for this study.

### 2.2. Lung function measures

Participants aged between 6 and 79 years were eligible for the spirometry test. The main exclusion criteria included recent chest pain and dyspnea, using supplemental oxygen, current surgery of the chest, abdomen and eye, and a recent stroke or heart attack. The test procedure complies with the recommendations of the American Thoracic Society (ATS). Each health technician received formal training. Expert consultants from the NIOSH Quality Control Center reviewed all the spirometry data collected. The lung function indices required for this study include FEV_1_, FVC, PEF, and FENO. We only use data with a quality grade of A and B to ensure the accuracy of the data. The Hankinson equation was used to translate the initial numbers to predicted percentages (16). The FENO data was calculated by averaging two reproducible FENO measurements. FEV_1_/FVC < 0.70 is considered an obstructive spirometry pattern and a restrictive spirometry pattern is defined by an FVC < 80% of predicted with FEV_1_/FVC ≥ 0.70. Further details can be accessed on the NHANES website.

### 2.3. Muscle quality index

Using dual-energy X-ray absorptiometry (DXA) data and handgrip strength (HGS) data from 2011 to 2012 in the NHANES database. Body composition data obtained from DXA scans included arm lean mass (kg) and leg lean mass (kg). It’s worth noting that lean mass from DXA includes both non-bone and non-fat tissue. ASM is defined as the sum of the lean mass of arms and legs (17). HGS was measured by a handgrip dynamometer. Participants who had no surgery and pain on either hand or wrist in the last 3 months can be eligible for the test. The grip test was performed in the standing position and participants were requested to squeeze the dynamometer as hard as possible. Each hand was tested three times, with 60secs between each test. The highest measurement of the hand was used. MQI (kg/kg) was defined as the ration between combined HGS divided by ASM (12). Information about DXA and HGS can be found on the NHANES website.

### 2.4. Covariate definitions

The potential covariates included age, sex, BMI, ethnicity, education level, the ration of family income to poverty (PIR) and cotinine. BMI was calculated as weight divided by height squared (kg/m^2^). We categorized education level as “no high school,” “high school graduate,” and “college or above.” The ration of family income to poverty (PIR) is an index of poverty status and it was classified into <1, 1–3, >3. Cotinine, a metabolite of nicotine, was used to assess the smoking status.

### 2.5. Statistical analysis

We used the recommended weighting scheme to analyze the data. All continuous variables were tested by the Shapiro–Wilk test. The continuous variables were expressed as a weighted mean and standard deviation or median and interquartile (IQR). Categorical variables were represented by the unweighted frequencies (weighted percentages). To compare characteristic differences between different MQI groups (quartiles), the weighted chi-squared and Kruskal-Wallis tests were used for categorical and continuous variables, respectively.

Multiple linear regression analyses were performed to analyze the relationship between the MQI and lung function indices between the four groups, and the lowest quartile group was the reference group. Besides, we treated the quartiles as continuous variables and performed a trend test (p for trend). Multivariable logistic regression was performed to analyze the relative risk ratio between MQI and different spirometry patterns, using the normal pattern as the reference group. We next used the restricted cubic splines to further show the association between MQI and lung function indices. Furthermore, we performed subgroup analysis based on whether the age was greater than 40 years. Age, sex, ethnicity, BMI, PIR, and cotinine were adjusted for in the model because we considered these covariates to be confounding factors associated with the study results. Lung function may be influenced by age, sex and ethnicity, etc. Cotinine represents an indicator of active and passive smoking and may better represent individual smoking status. In addition, BMI and PIR may influence MQI. All regressions are considered survey weights. All analyses were performed in R4.2.1 and two-tailed *p* < 0.05 was accounted for statistical significant.

## 3. Results

### 3.1. Baseline characteristics of study participants

A total of 1,588 participants were included in this study. The basic characteristics of the study population can be shown in Table 1. Among the 1,588 individuals, 839 were males and 719 were females. The weighted average age of the participants was 38 years. Most participants were non-Hispanic white, which accounted for 65%. 70% of individuals had a college degree or above. The media BMI was 27.1 (kg/m^2^), and the first quartile had the highest BMI relative to the other quartiles. There was no significant difference in cotinine intake among the four groups. Moreover, the media of FEV_1_/FVC, FVC%, FEV_1_%, PEF%, FENO were 80, 99, 97, 105, and 12 (ppb), respectively. A small number of participants (4.6%) had a restrictive pattern for spirometry.

**Table 1:** Baseline characteristics of study participants by muscle quality index quartiles (*N* = 1,588).

Characteristics	Total (1,558)	Q1 (445)	Q2 (354)	Q3 (370)	Q4 (389)	*p-*value
Age	38 (28, 48)	39 (28, 49)	39 (28, 49)	37 (28, 46)	36 (28, 49)	0.233
Gender						0.005
Male	839 (52%)	191 (41%)	185 (51%)	206 (54%)	257 (60%)	
Female	719 (48%)	254 (59%)	169 (49%)	164 (46%)	132 (40%)	
Ethnicity						<0.001
Mexican American	175 (9.2%)	46 (9.1%)	31 (6.6%)	41 (8.5%)	57 (12%)	
Other Hispanic	142 (6.9%)	32 (5.9%)	50 (9.7%)	33 (6.2%)	27 (5.8%)	
Non-Hispanic White	576 (65%)	148 (60%)	138 (69%)	148 (69%)	142 (64%)	
Non-Hispanic Black	381 (11%)	175 (21%)	77 (8.9%)	60 (7.0%)	69 (7.9%)	
Other	284 (7.3%)	44 (4.2%)	58 (5.9%)	88 (9.2%)	94 (9.9%)	
Education						0.346
No High school	233 (11%)	63 (11%)	50 (11%)	47 (8.8%)	73 (15%)	
High school graduate	303 (19%)	90 (21%)	65 (17%)	74 (18%)	74 (19%)	
College or above	1,022 (70%)	292 (68%)	239 (72%)	249 (73%)	242 (66%)	
Poverty-income ratio						0.252
<1	362 (17%)	127 (22%)	67 (13%)	80 (17%)	88 (15%)	
1–3	563 (32%)	161 (33%)	126 (33%)	129 (32%)	147 (32%)	
≥ 3	633 (51%)	157 (45%)	161 (54%)	161 (51%)	154 (53%)	
BMI (kg/m^2^)	27.1 (23.9, 31.3)	32.5 (27.6, 37.7)	28.3 (25.4, 31.6)	26.4 (23.6, 29.3)	24.1 (22.4, 26.8)	<0.001
Cotinine (ng/mL)	0.031(0.01,4.06)	0.033(0.01,3.42)	0.032 (0.01,6.87)	0.025(0.01,0.33)	0.039(0.01,46.30)	0.008
FENO (ppb)	12 (8, 18)	12 (8, 18)	12 (8, 19)	12 (10, 19)	12 (8, 17)	0.173
Spirometry						
FEV_1_/FVC	80 (76, 85)	81 (77, 85)	80 (76, 85)	80 (76, 83)	80 (77, 85)	0.046
FVC%	99 (91, 107)	96 (89, 105)	99 (91, 108)	100 (93, 108)	99 (92, 107)	0.030
FEV_1_%	97 (90, 106)	95 (88, 103)	98 (91, 107)	97 (90, 107)	98 (91, 105)	0.005
PEF%	105 (94, 116)	103 (92, 114)	105 (95, 118)	107 (95, 119)	105 (93, 117)	0.210
Spirometry pattern						0.194
Obstructive	97 (7.2%)	24 (6.0%)	16 (6.2%)	26 (8.4%)	31 (8.0%)	
Restrictive	108 (4.6%)	43 (7.7%)	27 (4.9%)	18 (3.4%)	20 (2.7%)	

FEV_1_%, percent-predicted FEV_1_; FVC%, percent-predicted FVC; PEF%, percent-predicted PEF; MQI, muscle quality index. Q1, the lowest MQI group (MQI ≤ 3.05 kg/kg); Q2, the lower MQI group (3.05–3.41 kg/kg); Q3, the higher MQI group (3.41–3.80 kg/kg); Q4, the highest MQI group (≥ 3.80 kg/kg).

### 3.2. Association between the MQI and lung function indices

Table 2 showed the association between the total MQI and lung function indices. In the unadjusted model, we did not find a correlation between PEF% and MQI; however, in the adjusted model, we were surprised to find a correlation between PEF% and MQI, and FVC% was always correlated with MQI in both the unadjusted and adjusted models. In addition, restrictive spirometry pattern lost its association with MQI in the adjusted model, which may be due to an inadequate sample size as patients with restrictive spirometry pattern only account for 1/15 of the total sample size in this study, which may have produced some error. A one-unit increase in MQI was related to higher percent predicted FVC (adjusted mean difference, 1.35; 95%CI, 0.10–2.59) and percent predicted PEF (adjusted mean difference, 1.92; 95%CI, 0.03–3.81).

**Table 2:** Association of muscle quality index with study outcomes.

	Unadjusted			Adjusted		
	MQI (Kg/Kg)	MQI per SD	*p-*value	MQI (Kg/Kg)	MQI per SD	*p-*value
Spirometry, MD (95CI%)						
FEV_1_/FVC	−0.07(−1.29, 1.14)	−0.04(−0.76, 0.67)	0.898	−0.27(−1.61, 1.06)	−0.16(−0.95, 0.63)	0.638
FEV_1_%	2.13(0.14, 4.12)	1.26(0.08, 2.43)	0.037	1.91(−0.19, 4.01)	1.13(−0.11, 2.37)	0.068
FVC%	2.21(0.44, 3.99)	1.31(0.26, 2.35)	0.018	2.28(0.17, 4.39)	1.35(0.10, 2.59)	0.038
PEF%	−0.16(−3.02, 2.70)	−0.09(−1.78, 1.59)	0.907	3.25(0.05, 6.45)	1.92(0.03, 3.81)	0.048
FENO (ppb)	0.19(−1.26, 1.65)	0.12(−0.74, 0.97)	0.780	−0.68(−2.54, 1.18)	−0.4(−1.50, 0.70)	0.407
Spirometry pattern, RRR (95CI%)						
Obstructive	1.13(0.69, 1.87)	1.08(0.80, 1.45)	0.621	0.91(0.49, 1.66)	0.94(0.66, 1.35)	0.751
Restrictive	0.53(0.31, 0.90)	0.68(0.50, 0.94)	0.019	0.58(0.03, 1.01)	0.72(0.52, 1.00)	0.053

MD, mean difference; RRR, relative risk ratio; FEV_1_%, percent-predicted FEV_1_; FVC%, percent-predicted FVC; PEF%, percent-predicted PEF. The adjusted models were adjusted by age, sex, BMI,ethnicity,poverty income ratio and cotinine.

Table 3 showed the relationship between MQI quartiles and lung function indices. Using MQI as a categorical variable and the first quartile as a reference, after model adjustment, FEV_1_% increased in Q2 and Q3 as MQI increased. We also found that after adjusting for confounding factors, FVC% increased only in Q3, with a mean difference of 4.22 (1.20, 7.23). It was obvious that PEF% increased in all quartiles and the value of p for the trend was 0.022. Frustratingly, neither FEV_1_/FVC nor FENO were found to be associated with MQI in each quartile. Moreover, in Q4, compared to normal individuals, higher MQI was related with a lower relative risk of a restrictive spirometry pattern in both the crude and adjusted models. (adjusted relative risk ratio, 0.34; 95% CI, 0.14–0.80).

**Table 3:** Effect of muscle quality index quartiles on lung function indices.

MQI (Kg/Kg)		Q1	Q2	Q3	Q4	P for trend
Unadjusted						
Spirometry, MD (95CI%)	
	FEV_1_/FVC	Ref	−0.26 (−1.18, 0.66)	−0.84 (−2.17, 0.50)	−0.24 (−1.89, 1.40)	0.601
	FEV_1_%	Ref	3.24 (0.34, 6.13)	3.28 (0.79, 5.78)	3.00 (0.20, 5.80)	0.034
	FVC%	Ref	3.05 (−0.44, 6.53)	3.88 (1.58, 6.18)	3.00 (0.06, 5.94)	0.026
	PEF%	Ref	4.49 (1.53, 7.46)	3.89 (0.07, 7.72)	0.81 (−3.19, 4.80)	0.761
	FENO(ppb)	Ref	−0.61 (−3.37, 2.16)	0.73 (−2.23, 3.70)	−0.06 (−2.98, 2.86)	0.788
Spirometry pattern, RRR (95CI%)
	Obstructive	Ref	1.02 (0.51, 2.04)	1.38 (0.67, 2.84)	1.3 (0.65, 2.63)	0.332
	Restrictive	Ref	0.62 (0.29, 1.31)	0.44 (0.18, 1.07)	0.34 (0.14, 0.81)	0.022
Adjusted						
Spirometry, MD (95CI%)
	FEV_1_/FVC	Ref	−0.10 (−1.52, 1.32)	−1.02 (−2.44, 0.39)	−0.39 (−2.52, 1.74)	0.406
	FEV_1_%	Ref	3.17 (0.01, 6.32)	3.01 (0.00, 6.02)	2.71 (−0.25, 5.66)	0.091
	FVC%	Ref	3.32 (−0.67, 7.32)	4.22 (1.20, 7.23)	3.16 (−0.72, 7.04)	0.065
	PEF%	Ref	5.19 (1.55, 8.83)	6.01 (2.14, 9.87)	5.43 (0.65, 10.22)	0.022
	FENO(ppb)	Ref	−1.47 (−5.21, 2.26)	−0.91 (−4.84, 3.02)	−1.31 (−5.36, 2.74)	0.511
Spirometry pattern, RRR (95CI%)
	Obstructive	Ref	0.75 (0.34, 1.63)	1.18 (0.58, 2.40)	0.88 (0.37, 2.09)	0.911
	Restrictive	Ref	0.64 (0.29, 1.42)	0.43 (0.16, 1.16)	0.34 (0.14, 0.80)	0.024

The adjusted models were adjusted by age, sex, BMI,ethnicity,poverty income ratio and cotinine. Q1, the lowest MQI group (MQI ≤ 3.05 kg/kg); Q2, the lower MQI group (3.05–3.41 kg/kg); Q3, the higher MQI group (3.41–3.80 kg/kg); Q4, the highest MQI group (≥ 3.80 kg/kg).

**Figure 2:**
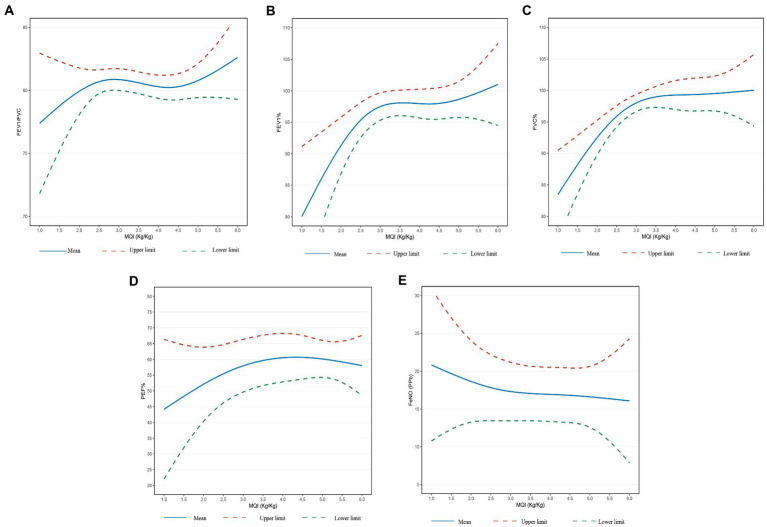
**(A)** Relationship between MQI and FEV_1_/FVC. **(B)** Relationship between MQI and FEV_1_% predicted. **(C)** Relationship between MQI and FVC% predicted. **(D)** Relationship between MQI and PEF% predicted. **(E)** Relationship between MQI and FENO. Adjusted for age, sex, BMI, ethnicity, poverty-income ratio and cotinine.

The results from the restricted cubic splines analysis were shown in Figure 2. Lung function indices increased significantly with increasing MQI and the relationships were in a non-linear trend. When the MQI was less than about 3.5 (kg/kg), the changes of lung function indices tended to increase in a linear trend and then gradually flat.

### 3.3. Subgroup analysis

Table 4 displayed the results of stratified analyses according to age. In the group of participants aged less than 40 years, we found no consistent evidence between MQI and lung function indices. In the group of participants aged over 40 years, in the unadjusted model, the three lung function indices (FEV_1_%, FVC%, and PEF%) increased with the increase of the MQI. And in the adjusted model, although FVC% reduced the relationship with MQI, the relationship between FEV_1_%, PEF% and MQI remained significant. The mean differences and 95% CI are 4.39 (1.09–7.69) and 7.02 (3.04–11.01), respectively. FEV_1_/FVC and FENO were not significantly associated with MQI in either of the two age groups. In addition, a lower MQI was associated with a higher relative risk of restrictive spirometry pattern (adjusted relative risk ratio, 0. 4; 95% CI, 0.17–0.96).

**Table 4:** Effect of muscle quality index on lung function indices, stratified by age.

Age,y	<40		≥40	
		Estimate (95%CI)	*p-*value	Estimate (95%CI)	*p*-value
Unadjusted					
Spirometry, MD (95CI%)
	FEV_1_/FVC	−0.5 (−1.43, 0.42)	0.27	−0.03 (−1.38, 1.32)	0.96
	FEV_1_%	−0.07 (−2.01, 1.86)	0.94	4.87 (2.48, 7.26)	<0.01
	FVC%	−0.12 (−1.86, 1.62)	0.88	5.18 (2.8, 7.55)	<0.01
	PEF%	−4.69 (−7.12, −2.26)	<0.01	5.78 (2.39, 9.16)	<0.01
	FENO(ppb)	0.77 (−1.77, 3.31)	0.53	−0.66 (−1.75, 0.42)	0.21
Spirometry pattern, RRR (95CI%)
	Obstructive	2.89 (1.54, 5.45)	<0.01	0.89 (0.51, 1.54)	0.68
	Restrictive	0.87 (0.47, 1.61)	0.66	0.36 (0.18, 0.71)	<0.01
Adjusted					
Spirometry, MD (95CI%)
	FEV_1_/FVC	−1.37 (−2.79, 0.06)	0.06	1.2 (−0.91, 3.3)	0.22
	FEV_1_%	0.54 (−1.78, 2.85)	0.60	4.39 (1.09, 7.69)	0.02
	FVC%	1.88 (0.24, 3.53)	0.03	3.38 (−0.04, 6.79)	0.05
	PEF%	0.69 (−2.43, 3.82)	0.62	7.02 (3.04, 11.01)	<0.01
	FENO(ppb)	−0.69 (−3.73, 2.35)	0.61	−0.56 (−2.03, 0.91)	0.40
Spirometry pattern, RRR (95CI%)
	Obstructive	2.01 (0.94, 4.28)	0.07	0.58 (0.31, 1.09)	0.09
	Restrictive	0.72 (0.43, 1.19)	0.19	0.4 (0.17, 0.96)	0.04

The adjusted models were adjusted by age, sex, BMI,ethnicity,poverty income ratio and cotinine.

## 4. Discussion

As far as we know, this was the first study to explore the connection among MQI and lung function indices in a sizeable and complex cohort of American adults. The results showed that as MQI rose, there seemed to be an increase in FEV_1_%, FVC%, and PEF%. Besides that, compared to individuals who have normal lung ventilation, the relative risk of a restrictive spirometry pattern was raised by a lower MQI. In addition, we found that the relationship between MQI and lung function indices was more significant in the higher age group compared to the lower age groups. Lung function is an important indicator of lung health, and when scores on a lung function test are below average, it is usually considered a lung disease. The reduced lung function in this study may reflect a loss of muscle mass and strength, which may be associated with sarcopenia.

MQI, a prominent component of sarcopenia, is regarded as a predictor of mortality and the probability of disability. In a study by Hu et al., it was demonstrated that asthma patients with sarcopenia, especially severe sarcopenia, had a much increased risk of airway obstruction and a significantly lower PEF (18). Our study also found that as MQI decreased, PEF% decreased significantly, however, we were unable to detect a higher risk of airway obstruction, which may be due to the varied populations that were selected for our investigation. Park et al. found that it is possible to identify healthy middle-aged people at high risk for rapid FEV_1_ reduction using the changes in body composition over time (19). This is basically consistent with our research results. We discovered that the correlation between MQI and lung function indices was better in Q3 compared to Q4, but their reference groups were all Q1, so we calculated P for trend and found *p* > 0.05, indicating that the effect of MQI on lung function was not linearly increasing, and also in the restricted cubic spline curve, which more intuitively proved to be non-linear, indicating MQI and lung function were in a non-linear relationship. We speculated that the reason for this non-linear trend may be that lung volume does not increase indefinitely with MQI in the population and there is a maximum limit to lung function, therefore the curve gradually flattens. BMI was highest in Q1 and no association was found between MQI and lung function in Q1, indicating that calculating height and weight alone is not representative of muscle mass. Additionally, several studies have demonstrated a connection between sarcopenia and deteriorated lung function and negative outcomes in chronic obstructive pulmonary disease (COPD) patients (5, 20).

In our research, we discovered a significant association between lung function decline and muscle loss in the middle-aged and older age groups. Such opinions have also been confirmed by a lot of research. Kera et al. concluded that PEF may be a reliable indication of sarcopenia using data from 427 older adults in the local community (4). Ridwan et al. further supported the view that PEF was independently associated with sarcopenia emergence in older people in a nationwide study (21). Also, in an older community-dwelling Korean population, Jeon et al. investigated a relationship between reduced muscle mass and low lung function, as indicated by lower FEV_1_ or FVC (22). All of these studies were consistent with our views that, after controlling for confounding variables, there was no significant relationship between MQI and lung function indices in the lower age groups. The higher age groups, however, showed the opposite result. Sarcopenia in older individuals has been used to categorize risk, predict negative outcomes, and trigger intervention targeted at avoiding deterioration in those most at risk (5). This is mainly because there is a gradual loss of muscle mass with age due to the progressive loss of motor neurons combined with a reduction in the quantity and size of muscle fibers. In some experimental models, specific intervention techniques have demonstrated positive outcomes for muscle loss and functional degeneration. In the clinic, if these or comparable therapies could benefit older people with sarcopenia by preserving muscle and enhancing mobility, there would be significant humanitarian advantages as well as financial savings for health care systems (23).

Some studies pointed out that in older adults, type 2 diabetes, chronic liver diseases (CLD), chronic kidney diseases (CKD), and cardiovascular diseases (CVD) are associated with accelerated loss of muscle strength and quality (24–26). Although some studies have not shown such a link, prevention of muscle loss remains important in middle-aged and older adults (21). This serves as a caution that muscle loss prevention is crucial for a variety of chronic conditions. Related studies have shown that resistance exercise (RE) can improve muscle mass, strength and physical performance (27, 28). Intake of protein, amino acids and vitamin D can also increase muscle mass and muscle strength (29, 30). In addition, a number of drugs have entered clinical trials to improve muscle mass through the administration of testosterone, selective androgen receptor modulators, myostatin inhibitors and growth hormone (31). For example, higher protein intakes at baseline were associated with less muscle strength loss over the period of the studies in the Women’s Health Initiative and the Framingham Offspring Cohort follow-up studies (32, 33). Visser et al. showed that the older adults with lower serum 25 (OH) D were twice as likely to have sarcopenia as those with higher serum 25 (OH) D (34). Another study proved resistance training can improve muscle mass and performance in older men (35). Even though some muscle deterioration is a natural part of aging, there are measures we can take to prevent the negative effects.

Our choice of predicted percentages instead of actual spirometry allowed us to reduce the interference of factors such as age, sex and height and is more suitable for the analysis of complex populations, which is one of our advantages over other studies. We chose cotinine instead of self-reported smoking status to represent smoking status and it reflects the overall biological effect of total smoking exposure. In addition, we integrated muscle mass as well as muscle strength to assess the muscle condition of the population, which is superior to those studies that just focused on muscle mass. We concur that MQI might not accurately represent sarcopenia as a whole because muscle function is implicated in sarcopenia. Our study did not, however, evaluate muscle function, which is an area we need to improve in the future. Although, according to the EWGSOP2, sarcopenia will be diagnosed in those who have low muscle mass or quality and low muscle strength, some published consensus by expert groups now include muscle function in the concept of sarcopenia (36). Also, our study could have missed some additional significant factors that might have affected the outcomes. Because of the small sample size in this study, we did not investigate whether other chronic diseases have an effect on lung function and muscle loss. A prospective study with a larger sample size and an all-age group may be done in the future. Finally, as this was a cross-sectional investigation, additional longitudinal cohort studies would be required to strengthen the temporal or causal correlation between MQI and lung function indices. Future multi-center, large-sample research will be necessary to confirm the findings presented above.

## 5. Conclusion

In conclusion, we found that MQI may associated with changes in lung function. Furthermore, in the middle-aged and older adult populations, lung function indicators and restrictive ventilation impairment were significantly associated with MQI, which seems to suggest that we should pay attention to muscle exercise in this group to improve lung function and avoid the development of chronic lung disease. This finding needs to be further confirmed by prospective future studies.

## Data availability statement

Publicly available datasets were analyzed in this study. This data can be found at: https://www.cdc.gov/nchs/nhanes/index.htm.

## Ethics statement

The NCHS Research Ethics Review Board examined and approved all research involving human subjects before they were used to gather data for the NHANES. Written informed permission was acquired by each subject. We conducted a study that was exempt from institutional review since it involves secondary data analysis from the NHANES.

## Author contributions

CC designed the study and revised the manuscript. LW and ZX was responsible for data collection, analysis, and manuscript writing. YC collected the data. All authors reviewed and approved the article’s submission.

## Funding

This study was financially supported by the Key Laboratory of Interventional Pulmonology of Zhejiang Province (2019E10014), the Zhejiang Provincial Key Research and Development Program (2020C03067), and the National Nature Science Foundation of China (82170017).

## Conflict of interest

The authors declare that the research was conducted in the absence of any commercial or financial relationships that could be construed as a potential conflict of interest.

## Publisher’s note

All claims expressed in this article are solely those of the authors and do not necessarily represent those of their affiliated organizations, or those of the publisher, the editors and the reviewers. Any product that may be evaluated in this article, or claim that may be made by its manufacturer, is not guaranteed or endorsed by the publisher.
